# Correction: Theoretical calculation of a full-dimensional *ab initio* potential energy surface and prediction of infrared spectra for Xe–CS_2_

**DOI:** 10.1039/c9ra90055a

**Published:** 2019-07-22

**Authors:** Miao Qin, Xiuchan Xiao, Hua Zhu

**Affiliations:** School of Architectural and Environmental Engineering, Chengdu Technological University Chengdu 611730 China shawailsa@sina.cn; Center of Big Data for Smart Environmental Protection, Chengdu Technological University Chengdu 611730 China; School of Chemistry, Sichuan University Chengdu 610064 China

## Abstract

Correction for ‘Theoretical calculation of a full-dimensional *ab initio* potential energy surface and prediction of infrared spectra for Xe–CS_2_’ by Miao Qin *et al.*, *RSC Adv.*, 2019, **9**, 20925–20930.

The authors regret that eqn (4) was displayed incorrectly in the PDF version of the original article. The correct version of eqn (4) is presented below:
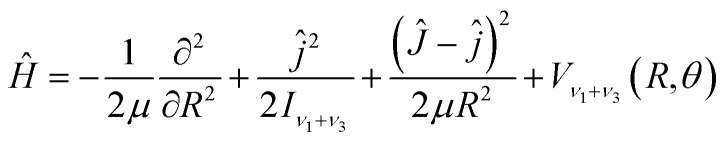


The Royal Society of Chemistry apologises for these errors and any consequent inconvenience to authors and readers.

## Supplementary Material

